# The Daily Mile and children’s physical activity, mental health and educational performance: a quasi-experimental study in Greater London primary schools

**DOI:** 10.1136/bmjsem-2025-002821

**Published:** 2026-01-03

**Authors:** Bina Ram, Mark Cunningham, Emanuela Falaschetti, Anna Chalkley, Thomas Woodcock, Esther van Sluijs, Dougal Hargreaves, Sonia Saxena

**Affiliations:** 1 Primary Care and Public Health, Imperial College London School of Public Health, London, UK; 2 Clinical Trials Unit, Imperial College London Faculty of Medicine, London, UK; 3 University of Bradford Faculty of Health Studies, Bradford, UK; 4 Centre for Applied Education Research, Wolfson Centre for Applied Health Research, Bradford Royal Infirmary, Bradford, UK; 5 MRC Epidemiology Unit, University of Cambridge School of Clinical Medicine, Cambridge, UK; 6 Mohn Centre for Children’s Health and Wellbeing, Imperial College London School of Public Health, London, UK

**Keywords:** Health promotion, Physical activity, Public health

## Abstract

**Objectives:**

To examine associations between The Daily Mile, a school-based active mile intervention, and pupils’ physical activity, mental health and educational performance.

**Methods:**

Year 1 pupils (aged 5–6 years) from Greater London primary schools were invited. Schools were allocated to The Daily Mile or non-Daily Mile group based on their reported Daily Mile participation. We measured weekday school hours mean minutes of moderate-to-vigorous physical activity (MVPA) using GENEActiv accelerometers. Other outcomes included time spent sedentary and in light activity, mental health and educational performance. Multi-level linear regression models examined differences accounting for repeated measurements (day) clustered by pupils-, class- and school-level, with adjustments for sex, ethnic group, area-level deprivation and month of assessment.

**Results:**

A total of 1004 pupils/40 schools were recruited and assessed between October 2021 and January 2023; 21 schools/499 pupils comprised The Daily Mile group; 19 schools/505 pupils the non-Daily Mile group. Daily Mile pupils spent 2.2 min more in MVPA compared with non-Daily Mile pupils, but the difference was not significant (0.78, 95% CI −2.14 to 3.69). Daily Mile pupils spent less time sedentary and more time in light activity compared with the non-Daily Mile pupils, but not significantly (−5.06, 95% CI −15.37 to 5.26 and 3.27, 95% CI −4.26 to 10.81, respectively). There were no differences in mental health or educational performance.

**Conclusions:**

We found no associations between The Daily Mile and pupils’ physical activity, mental health and educational performance. Pupils in our study were in year 1 with early exposure to the intervention; assessments over longer periods are needed to understand any benefits.

**WHAT IS ALREADY KNOWN ON THIS TOPIC**

- Less than half of children in England meet national guidelines of 60 min of moderate-to-vigorous physical activity (MVPA) per day, 30 min of which should take place during the school day.
- The Daily Mile, a school-based active mile intervention, is recommended by UK Government policy as an easy and accessible approach to increase children’s physical activity.

**WHAT THIS STUDY ADDS**

- Our study is among the first large-scale studies to examine associations between engaging in The Daily Mile on pupils’ health and educational performance, in a diverse real-world urban conurbation.
- Our findings show that during the school day, Daily Mile pupils on average spent 2 min more in MVPA, more time in light activity and less time sedentary compared with non-Daily Mile pupils. There were no adverse effects on children’s health or educational performance.

**HOW THIS STUDY MIGHT AFFECT RESEARCH, PRACTICE OR POLICY**

- Interventions such as The Daily Mile require sustainability and longer-term follow-up to identify any beneficial impacts.

## Introduction

 In the UK, less than half (47%) of children aged 5–16 years meet the recommended 60 min of daily moderate-to-vigorous physical activity (MVPA),^1^ of which 30 min should take place during the school day.^2^ Engaging in physical activity (PA) has been associated with improved physical fitness, bone health, mental health and cognition, and can help reduce the symptoms of depression.^3^ Regular PA during childhood can lead to better health and well-being in adulthood.^4^ However, physical inactivity among children remains high in the UK and disparities exist; those from lower-income households, some ethnic minority groups and girls compared with boys are less likely to be physically active.^1 5^

The Daily Mile is a school-based active mile intervention widely promoted in PA health policy^6^,^8^ to help children meet PA targets.^9^ The intervention aims to provide children with MVPA during the school day, in addition to their regular PA and is reported to make children healthier, happier and perform better in class.^10^ The Daily Mile was recommended in the UK Government Childhood Obesity Strategy in both 2016 and 2018.^6 7^ The Scottish Government encouraged Daily Mile uptake in 2017^8^ and continues to endorse The Daily Mile across all Scottish local authorities.^11^ Over the past decade, uptake of The Daily Mile has increased globally; 21 237 schools and nurseries in 98 countries were registered as of December 2024.^12^ In England, one in five primary schools is registered, with reach to schools in urbanised areas and those with higher levels of need (ie, pupils from lower-income households).^13^

The Daily Mile’s grassroots popularity and uptake in schools suggest it is a feasible and equitable intervention. Previous experimental studies have reported that children accumulate between 5 and 15 min of MVPA, increased fitness and reductions in sedentary time during the adoption phase of The Daily Mile.^14 15^ Pragmatic trial evidence has shown The Daily Mile to be cost-effective in increasing PA in primary school-aged children but limited evidence of impact on adiposity.^15 16^ Qualitative evidence suggests a positive impact on children’s motivation and self-esteem^17 18^ and academic performance.^14 19^ However, despite qualitative studies reporting improvements to children’s mental health and educational performance, there is limited or inconsistent quantitative evidence of either.^14 16 19^ There is an increasing number of published evaluations of The Daily Mile, but large-scale studies are lacking, and the mixed evidence base of the effects in real-world settings remains to be established.

iMprOVE is a quasi-experimental observational study of pupils in a real-world urban multi-ethnic setting of Greater London primary schools.^20^ This paper presents the cross-sectional results from the first wave of data collection. The primary aim of this study was to compare pupils’ MVPA minutes during the school day in schools doing The Daily Mile compared with schools that are not. Our hypothesis is that pupils in schools that do The Daily Mile will spend more minutes in MVPA during school hours on weekdays compared with pupils in schools that do not do The Daily Mile. Our secondary outcomes examined associations between The Daily Mile and mental health, and educational performance.

## Methods

### Study design, participants and data

Using a quasi-experimental design study, recruitment and data collection for iMprOVE took place over a 16-month period (October 2021–January 2023). Our aims in wave 1 were to recruit 1000 children in year 1 (aged 5–6 years) from Greater London state-funded primary schools.^20^ Schools completing our previous survey^21^ provided a sampling frame to recruit schools into this study. To increase participation rates and to ensure geographical representation across London boroughs, we selected primary schools outside our sampling frame (ie, schools in London that did not respond to our survey).

Schools were invited to participate by email; those responding with an interest were contacted by phone. Written consent was obtained from the schools, and the study team visited the schools to speak to and distribute information to pupils (study information sheets and consent/assent forms). The parent/guardian and their child were required to consent to participate in the study. Completed forms were returned to the schools and collected by a study researcher. A convenient date and time were arranged to take measures of each participating pupil’s bio-impedance and issue them with an accelerometer. Verbal instructions were given for wearing the monitor, the duration of wear, and when and how to return the monitors. An information pack was given to the pupils, which included monitor wear instructions and questionnaires for the parent/guardian and pupil to complete. Teachers were given a questionnaire to complete for each participating pupil. After the wear period, accelerometers and questionnaires were returned to the school and collected by a study researcher. Each pupil was sent a certificate and £5 Amazon voucher as a thank you for returning the accelerometer and questionnaires.

### Intervention

The Daily Mile is a whole-school approach to increase pupils’ PA by running or jogging for 15 min in addition to their regular PA during the school day.^10^ Though not compulsory, UK health policy recommends it as a free, easy and accessible way for schools to help pupils meet PA targets.^6^,^911^

We used class teacher responses to the question ‘Does your school take part in The Daily Mile?’ (included in the teacher questionnaire) to assign schools to The Daily Mile or non-Daily Mile group. In instances where there was a difference in reporting Daily Mile participation across year 1 classes within the same school, we assigned the school to The Daily Mile group, assuming that pupils would be exposed to the intervention at school level.

### Outcomes and measures

Our outcomes and measures have been described in detail elsewhere.^20^ In brief, our primary outcome was mean minutes of MVPA during school hours on weekdays, measured using the GENEActiv (ActivInsights, Kimbolton, UK) wrist-worn accelerometers. Acceleration was sampled at 85.70 Hz, for a consecutive full 7-day wear period. We used the GGIR R-package^22 23^ to process data recorded by the accelerometers by applying 5 s epochs to total activity minutes. We defined PA intensity (sedentary, light, MVPA) based on thresholds reported by Phillips *et al*.^24^ As The Daily Mile is a school-based intervention, we included PA data for weekdays and school hours only. Start and end times for each school were included in the GGIR data processing methods; therefore, PA for each pupil is based on their school’s exact school hours/minutes. To make use of all valid data in our analysis, pupils who did not provide at least one full school day of data were excluded.

We examined three secondary outcomes: (1) all day activity in different intensities (sedentary, light, MVPA) and segmented by before, during and after school on weekdays; (2) mental health (using the validated Strengths and Difficulties Questionnaire (SDQ)^25^) reported by parents; and (3) educational performance (UK’s National Curriculum’s ‘age related expectations’ for reading, writing and maths^26^) reported by teachers. For the SDQ, we used the tool’s guidelines to calculate a ‘total difficulties’ score for each child with a minimum score of 0 and maximum score of 40 (higher scores indicating more difficulties). Ratings for educational performance were on a 5-point Likert scale ranging from 1 (below expected levels) to 5 (above expected levels).^16^

In addition to our primary and secondary outcomes, we measured pupils’ anthropometry (Tanita DC-240 MA Body Composition Analyser; Tanita; Tokyo, Japan). Height was measured to the last complete millimetre with a portable stadiometer (Seca, Leicester). Body mass index (BMI) was calculated as weight (kg)/height (m^2^). We used the British 1990 growth reference (UK90) to assign weight status for each child according to their BMI as underweight (<2nd centile), healthy weight (>2nd to <85th centile), overweight (>85th to <95th centile) or very overweight (>95th centile).^27^

We accessed online, freely accessible data to obtain school characteristics including type of school (Academy Converter, Academy Sponsor Led, Free Schools); school size, that is, total pupil numbers; pupil gender; number of pupils with special educational needs and disabilities; number of pupils with English as a second language; number of pupils eligible for free school meals and Ofsted rating (Ofsted is the UK’s ‘Office for Standards in Education, Children’s Services and Skills’).^28^,^30^

### Covariates

We collected data for a priori hypothesised covariates; demographic data of pupils, including sex (male/female) and ethnic group (white, mixed/multiple, Asian, black and other) reported by the parent/guardian. Area-level deprivation (based on school postcode) using the Income Deprivation Affecting Children Index (IDACI),^31^ which represents the proportion of children (aged under 16 years) in income-deprived families. We grouped IDACI scores into quintiles; lower levels indicating schools in areas with the most deprivation. To allow for seasonal effects of engaging in PA, the month of assessment was also included as a covariate in our analyses.

### Statistical analysis

We compared differences in school-level characteristics between recruited schools and non-recruited schools. We also compared differences in school- and pupil-level characteristics of those allocated to the Daily Mile and non-Daily Mile groups. To assess differences between groups, we used Pearson’s χ^2^ tests for categorical variables and independent samples t-test or Wilcoxon rank (Mann-Whitney) for continuous variables. Means or medians and IQRs are presented accordingly.

For our main analysis, participants with valid data for each outcome were included. We used a four-level mixed-effects linear regression model accounting for repeated (daily) measurements, clustered within pupils, pupils within classes and classes within schools as random effects, and covariates as fixed effects to estimate differences in mean minutes of MVPA during school hours between the Daily Mile and non-Daily Mile groups. Our mixed model approach allowed for the assumption of missing at random. MVPA was included as a continuous variable, and all covariates (sex, ethnic group, IDACI and month of assessment) were included as categorical. Unadjusted (differences in mean minutes of MVPA allowing for random effects) and fully adjusted (all covariates) models are presented with the 95% CI. We used the Stata post-estimation command *predict* to calculate means and SD of minutes of MVPA to allow for clustering. Regression models were repeated for each secondary outcome in separate regression models. However, for SDQ and educational performance outcomes, we removed the random effects in the unadjusted model and the fixed effect of month of assessment in the fully adjusted model. We carried out sensitivity analyses on our primary outcome by: (1) excluding schools where classes varied in Daily Mile participation; and (2) including BMI as a covariate in our model (as BMI is a strong predictor of MVPA^32 33^). All statistical analyses were conducted using Stata V.18 (https://www.stata.com/company/). We report our findings in line with the Strengthening the Reporting of Observational Studies in Epidemiology guidelines (online supplemental material 1).^34^

### Patient and public involvement

We consulted with our Steering Group throughout our study, which included professional and public representatives from The Daily Mile Foundation, academic researchers and representatives from Sport England, London Marathon and London Sport, and parents and children, who were involved in the piloting of questionnaires and assessments, and dissemination of our work.

## Results

### Schools, participants and data

**Figure 1:**
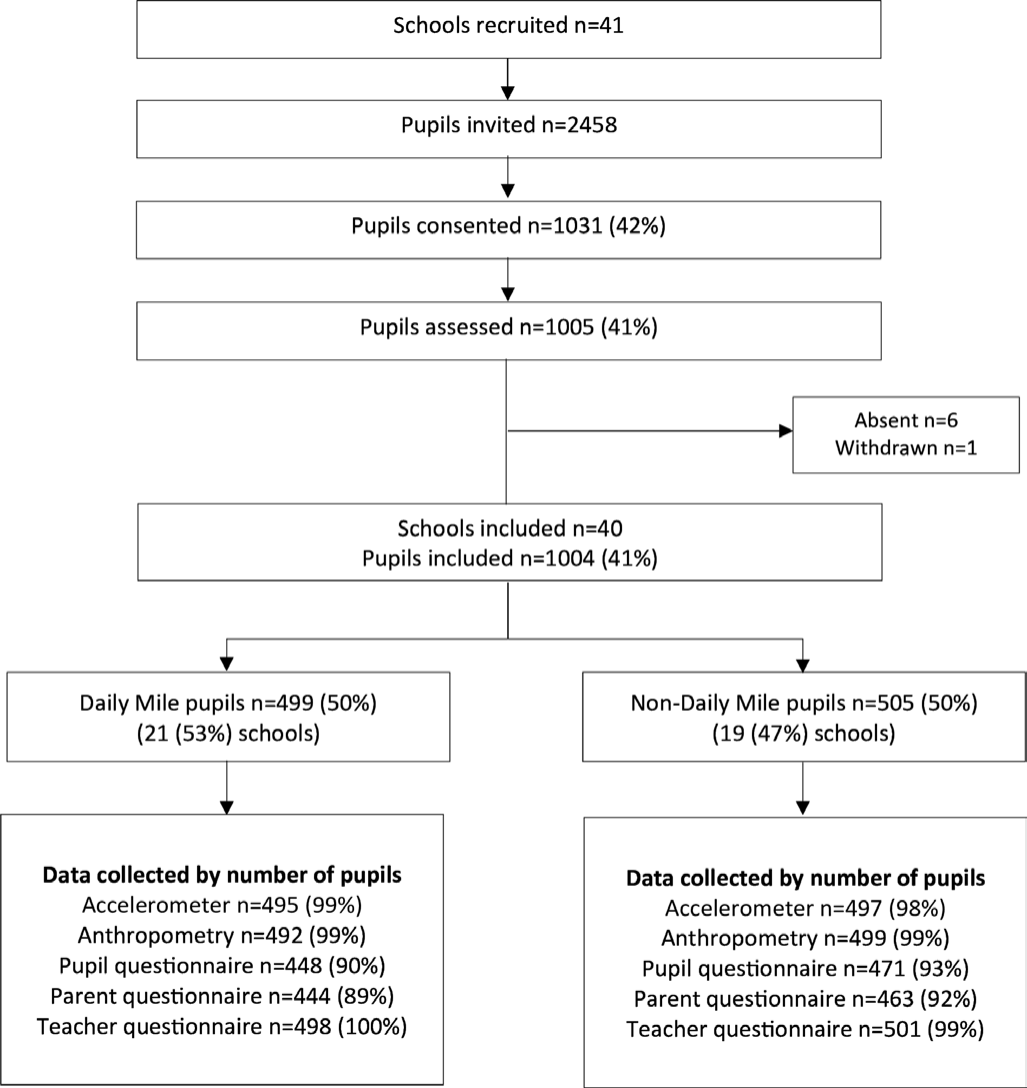
iMprOVE study recruitment and data.

A total of 41 schools were recruited, from which 2458 year 1 pupils were invited to participate; 1005 (41%) consented and were assessed (figure 1). One school withdrew after consent; therefore, 40 schools participated in the study (n=1004 pupils). Classes within four schools differed in their responses to doing The Daily Mile. Our sample had 21 schools with 499 (50%) pupils in the Daily Mile group, and 19 schools with 505 (50%) pupils in the non-Daily Mile group. Figure 1 shows the study recruitment and data collected.

### School characteristics

Schools recruited into the study were broadly representative of schools in London. However, the number of pupils with English as a second language was significantly lower compared with non-participating schools (13% vs 30%, respectively, p=0.02; online supplemental material 2). Among the schools participating, there were no characteristic differences between the Daily Mile and non-Daily Mile schools (table 1).

**Table 1:** Characteristics of Daily Mile and non-Daily Mile schools

Schools, n (%*)	Daily Mile group†	Non-Daily Mile group†	All	P value (Daily Mile vs non-Daily Mile group)
	21 (53)	19 (48)	40 (100)	
Type of school‡, n (%*)				
Academy/free	3 (14)	2 (11)	5 (13)	1.00§
Local authority	18 (86)	17 (89)	35 (88)	
School size (total pupil numbers), n (%*)				
≤500	14 (67)	16 (84)	30 (75)	0.28§
>500	7 (33)	3 (16)	10 (25)	
School pupil sex, n (%*)				
Mixed	21 (100)	19 (100)	40 (100)	–
Non-mixed	0	0	0	
Pupils with SEND, n (%*)				
≤100	20 (95)	18 (95)	38 (95)	1.00§
>100	1 (5)	1 (5)	2 (5)	
Pupils with English as second language, n (%*)				
≤100	3 (14)	2 (11)	5 (13)	1.00§
>100	18 (86)	17 (89)	35 (88)	
Pupils eligible for free school meals, n (%*)				
≤100	13 (62)	13 (68)	26 (65)	0.67¶
>100	8 (38)	6 (32)	14 (35)	
Ofsted rating**, n (%*)				
Outstanding/good	18 (86)	17 (89)	35 (88)	1.00§
Other	3 (14)	2 (11)	5 (13)	
Area deprivation IDACI (quintiles), n (%*)				
(Most deprived) 1	1 (5)	4 (21)	5 (13)	0.24§
2	7 (33)	4 (21)	11 (28)	
3	6 (29)	6 (32)	12 (30)	
4	2 (10)	4 (21)	6 (15)	
(Least deprived) 5	5 (24)	1 (5)	6 (15)	

* % may not total 100 due to rounding.

† Schools in the Daily Mile group are those where at least one participating class per school reported doing the Daily Mile. Non-Daily Mile group are schools where none of the schools reported doing the Daily Mile.

‡ Academy/free schools are academy converter, academy sponsor-led and free schools; local authority schools are community, foundation, voluntary aided and voluntary controlled.

§ Fisher’s exact test.

¶ χ2 test.

** Ofsted: The Office for Standards in Education, Children's Services and Skills; ‘other’ includes ‘requires improvement’, ‘inadequate’ (rated ‘serious weaknesses’ or ‘special measures’) or rating not available.

IDACI, Income Deprivation Affecting Children Index; SEND, Special Educational Needs and Disabilities.

### Pupil characteristics

Pupils’ mean age was 6.1 years (SD 0.4). We found no differences in characteristics between pupils included in the Daily Mile and non-Daily Mile groups (table 2).

**Table 2:** Pupil characteristics by Daily Mile and non-Daily Mile groups

	Daily Mile group	Non-Daily Mile group	All	P value (Daily Mile vs non-Daily Mile pupils)
Pupils recruited, n (%)	**499** (**50**)	**505** (**50**)	**1004** (**100**)	
Sex, n (%)				
Male	255 (51)	264 (52)	519 (52)	0.71*
Female	244 (49)	241 (48)	485 (48)	
Ethnic group, n (%)	**n=414**	**n=429**	**n=843**	
White	162 (32)	183 (36)	345 (34)	0.34*
Mixed/multiple	52 (10)	66 (13)	118 (12)	
Asian	137 (27)	119 (24)	256 (26)	
Black	47 (9)	49 (10)	96 (10)	
Other	16 (3)	12 (2)	28 (3)	
General health status, n (%)	**n=451**	**n=473**	**n=924**	
Very good/good	438 (88)	459 (91)	897 (97)	1.00†
Fair	12 (2)	13 (3)	25 (3)	
Bad/very bad	1 (<0)	1 (<0)	2 (<0)	
Limiting health conditions, n (%)	**n=447**	**n=467**	**n=914**	
Yes	18 (4)	26 (5)	44 (4)	0.28*
Anthropometry, mean (SD**)**	**n=492**	**n=499**	**n=991**	
BMI‡	16.0 (2.2)	16.0 (2.1)	16.0 (2.2)	0.93§
Fat %	21.1 (5.4)	20.5 (5.2)	20.8 (5.3)	0.08§
Weight¶, n (%)				
Underweight	30 (6)	32 (6)	62 (6)	0.48*
Healthy weight	381 (77)	394 (79)	775 (78)	
Overweight	28 (6)	33 (7)	61 (6)	
Obese	53 (11)	40 (8)	93 (9)	
Days of valid physical activity data, n (%)	**n=460**	**n=471**	**n=931**	
5 days	382 (83)	386 (82)	768 (82)	0.93*
4 days	47 (10)	52 (11)	99 (11)	
3 days	12 (3)	16 (3)	28 (3)	
2 days	13 (3)	11 (2)	24 (3)	
1 day	6 (1)	6 (1)	12 (1)	

* χ2 test.

† Fisher’s exact test.

‡ BMI calculated as weight (kg)/height (m2).

§ t-test.

¶ Weight categories based on British 1990 Growth Reference (UK90) centiles.

BMI, body mass index.

### Outcomes

Each child contributed a mean 4.7 (SD: 0.76) days of valid data. During school hours, Daily Mile pupils spent an average of 2.2 min more in MVPA (mean 11.4; SD 11.5) compared with the non-Daily Mile group (mean 9.2; SD 9.4). After multi-level linear regressions, we found no significant differences in mean minutes of MVPA between the two groups during school hours on weekdays. The differences remained non-significant in the fully adjusted model (table 3). In our exploratory subgroup analyses by covariate, IDACI quintile showed a statistically significant difference (11.16; 95% CI 0.42 to 21.89; table 3). However, due to the number of comparisons made, this finding may be likely due to chance and should be interpreted with caution.

**Table 3:** Difference in mean minutes of MVPA, Daily Mile group compared with the non-Daily Mile group

	Daily Mile group (n=460)	Non-Daily Mile group (n=471)	Model 1* (unadjusted) Difference (95% CI)	Model 2† (adjusted) Difference (95% CI)
	Mean (SD)‡	Mean (SD)‡	(Reference=non-Daily Mile group)	
MVPA minutes§	11.4 (11.5)	9.2 (9.4)	1.68 (−1.17 to 4.53)	0.78 (−2.14 to 3.69)
Sex (reference=females)				1.15 (−0.07 to 2.37)
Ethnic group (reference=white)				
Mixed				0.90 (−0.97 to 2.78)
Asian				1.74 (−0.00 to 3.48)
Black				0.07 (−2.18 to 2.31)
Other				2.14 (−1.41 to 5.69)
IDACI quintiles (reference=1 (most deprived) borough)				
2				7.60 (−2.13 to 17.33)
3				6.82 (−3.21 to 16.84)
4				2.48 (−8.10 to 13.05)
5 (least deprived)				11.16 (0.42 to 21.89)¶
Month of assessment** (reference=January)				
February				−1.93 (−11.78 to 7.92)
March				1.00 (−8.53 to 10.53)
April				−2.63 (−15.05 to 9.79)
May				0.02 (−9.23 to 9.28)
June				0.97 (−8.17 to 10.11)
July				4.37 (−8.01 to 16.74)
October				17.24 (−2.55 to 37.02)
November				−2.18 (−11.80 to 7.43)
December				4.69 (−8.62 to 18.09)

* Model 1 accounts for repeated measures by day and random effects for clustering at pupil, class and school level.

† Model 2 includes model 1+adjustments for sex, ethnic group, IDACI and month of assessment.

‡ Postestimation command *predict* used to determine mean and SD.

§ Total MVPA minutes and number of days per child with full school hours data.

¶ Significant at p<0.05 level.

** No assessments took place during the months of August or September.

IDACI, Income Deprivation Affecting Children Index; MVPA, moderate-to-vigorous physical activity.

The Daily Mile group spent less time being sedentary compared with the non-Daily Mile group (mean 338.79, SD 45.9 vs mean 349.33; SD 40.8), and more time in light activity (mean 40.12, SD 3.9 vs 32.95, SD 28.0) during the school day. However, these differences were not significant (table 4).

**Table 4:** Different intensities of physical activity on weekdays, Daily Mile group compared with the non-Daily Mile group

Weekday physical activity (min)	Daily Mile group	Non-Daily Mile group	Model 1* (unadjusted)	Model 2† (adjusted)
	n=460	n=471	Difference (95% CI)	Difference (95% CI)
	Mean (SD)‡	Mean (SD)‡	(Reference=non-Daily Mile group)	
All day§				
Sedentary	1016.01 (71.2)	1006.74 (76.8)	5.25 (−14.84 to 25.34)	1.70 (−12.18 to 15.58)
Light	317.42 (46.7)	322.2 (48.6)	−2.84 (−15.86 to 10.17)	0.60 (−8.43 to 9.62)
MVPA	106.58 (28.5)	111.05 (31.8)	−1.88 (−9.51 to 5.76)	−2.26 (−7.79 to 3.27)
Before school¶				
Sedentary	258.55 (53.8)	249.37 (58.3)	3.59 (−13.37 to 20.55)	8.94 (−7.27 to 25.16)
Light	197.82 (36.5)	202.37 (37.6)	−1.64 (−12.86 to 9.59)	−4.22 (−14.65 to 6.21)
MVPA	70.40 (21.6)	74.32 (24.2)	−0.89 (−7.65 to 5.88)	−3.51 (−9.74 to 2.72)
During school**				
Sedentary	338.79 (45.9)	349.33 (40.8)	−6.91 (−16.90 to 3.08)	−5.06 (−15.37 to 5.26)
Light	40.12 (33.9)	32.95 (28.0)	4.98 (−2.23 to 12.20)	3.27 (−4.26 to 10.81)
MVPA	11.4 (11.5)	9.2 (9.4)	1.68 (−1.17 to 4.53)	0.78 (−2.14 to 3.69)
After school††				
Sedentary	418.38 (69.6)	407.75 (66.3)	1.40 (−10.79 to 13.59)	−4.22 (−16.40 to 7.97)
Light	79.46 (50.4)	86.9 (46.40)	−0.17 (−9.51 to 9.17)	3.87 (−5.53 to 13.27)
MVPA	24.70 (19.8)	27.50 (18.9)	−1.16 (−5.30 to 2.97)	0.91 (−3.13 to 4.95)

* Model 1 accounts for repeated measures by day and random effects for clustering at pupil, class and school level.

† Model 2 includes model 1+adjustments for sex, ethnic group, Income Deprivation Affecting Children Index and month of assessment.

‡ Postestimation command *predict* used from model 1 to determine mean (SD) difference in physical activity minutes between the Daily Mile and non-Daily Mile groups.

§ All day activity includes 24-hour period for school days.

¶ Before school measured from midnight to school start time per school.

** During school MVPA as presented in table 3.

†† After school measured from per school end time to midnight.

MVPA, moderate-to-vigorous physical activity.

Mental health and educational performance were similar and not significant for both groups (table 5).

**Table 5:** Differences in mental health and educational performance, Daily Mile group compared with the non-Daily Mile group

		Daily Mile group	Non-Daily Mile group	Model 1 (unadjusted)	Model 2* (fully adjusted)
		Mean (SD)†	Mean (SD)†	Difference (95% CI)	Difference (95% CI)
				(Reference=non-Daily Mile group)	
Mental health (SDQ)‡		**n=435**	**n=459**		
		8.5 (0.5)	8.8 (0.6)	−0.25 (−1.09 to 0.58)	−0.52 (−1.28 to 0.23)
Educational performance§	Reading	**n=497**	**n=495**		
		3.3 (0.2)	3.3 (0.2)	−0.01 (−0.23 to 0.21)	0.00 (−0.20 to 0.21)
	Writing	**n=495**	**n=496**		
		3.1 (0.2)	3.1 (0.2)	0.01 (−0.18 to 0.20)	0.06 (−0.14 to 0.25)
	Maths	**n=494**	**n=495**		
		3.3 (0.2)	3.4 (0.2)	−0.05 (−0.25 to 0.15)	0.00 (−0.20 to 0.21)

* Fully adjusted model 2 includes adjustments for sex, ethnic group and Income Deprivation Affecting Children Index.

† Postestimation command *predict* used from model 1 to determine mean (SD) difference in physical activity minutes between the Daily Mile and non-Daily Mile groups.

‡ Mental health as measured by the Strengths and Difficulties Questionnaire (SDQ) reported by parents. Total difficulties score is the sum of scores on 4/5 SDQ subscales; scores range from 0 to 40.

§ Age related expectations; reported by class teacher; scale from 0 (below expected) to 4 (above expected).

n, number of children with complete data.

### Sensitivity analysis

Findings from both sensitivity analyses were consistent with the main analyses. There were no significant differences in mean minutes of MVPA between the Daily Mile group and non-Daily Mile group in both the unadjusted and fully adjusted models when we: (1) excluded four schools which reported a difference among classes participating in The Daily Mile (online supplemental material 3); and (2) included BMI percentiles as a covariate in our fully adjusted model, despite a small positive effect on MVPA (0.02; 95% CI 0.00 to 0.04; online supplemental material 4).

## Discussion

Our study is among the first to examine Daily Mile associations with health and educational outcomes in a representative study population of primary schools in a real-world urban setting. Although our findings show that during the school day, Daily Mile pupils spent more time in MVPA and light activity, and less time sedentary compared with non-Daily Mile pupils, our results did not find any significant differences between the two groups.

Our findings support those of a Cochrane review of school-based PA interventions^35^ which showed that PA interventions aimed at schools have little-to-no impact on time spent in MVPA or in sedentary time.^35^ Previous studies have used randomised control trials (RCTs) and quasi-experimental designs to examine the impact of The Daily Mile on health outcomes. An RCT study in Birmingham compared BMI z scores of pupils engaging in and not engaging in The Daily Mile and found no differences in their outcomes of fitness and psychological well-being.^16^ Though our outcomes differed, our results support these findings. However, our findings differed from that of another RCT which found that aerobic fitness significantly increased among pupils doing The Daily Mile.^36^ Quasi-experimental studies have reported significant differences in pupils’ increased MVPA (9 min per day), and significant reductions in sedentary time (18 min per day) in the schools which did The Daily Mile compared with those that did not,^15^ and in significantly increased cardiorespiratory fitness.^37^ Overall, findings from both RCTs and quasi-experimental studies evaluating The Daily Mile have been inconsistent, which contributes to inconclusive intervention effects. However, the advantage of quasi-experimental designs offers insights into real-world implementation which may facilitate or hinder the effectiveness of the intervention. Moreover, a systematic review of studies assessing The Daily Mile on children’s outcomes published in 2023 concluded that there is evidence to suggest that The Daily Mile can increase children’s PA and fitness, but existing evidence lacks high-quality research and highlights the need for follow-up to establish sustainability and life-long impacts.^38^ In our study, pupils were in the first year of primary school after prolonged COVID-19 lockdowns and school closures during which PA had reduced and mental health worsened. One London-based study found that children of key workers who continued to attend school during lockdowns maintained higher PA levels and that loss of in-person schooling had the single largest impact on reducing pupils’ PA.^39^ Hence, there is plausible evidence that school-based interventions such as The Daily Mile can contribute to increasing children’s PA.

Our study’s strengths are its size and inclusion of a representative primary school population in a real-world setting which furthers understanding of potential intervention effects outside of a ‘research setting’. The schools included were from an ethnically diverse urban conurbation and data accuracy and completeness were to a high standard, including the use of individual-level objectively measured PA data. The use of wearable digital devices for assessments of PA is rapidly evolving. Achieving an 82% 5-day PA measure compliance rate of children aged 5–6 years provided robust estimates of PA compared with self-reported PA, minimising potential participant bias of overestimating or underestimating time being active.^1^ The use of validated questionnaires ensured consistent and reliable data collection, thus reducing bias. Our statistical analysis accounted for the hierarchical nature of the data and adjusted for SE estimates for the impact of clustering. However, as we did not adjust for multiplicity for exploratory secondary outcomes, this increases the risk of a type I error. Though our findings may be potentially generalisable to other diverse urban conurbations, this should be considered with caution due to the self-selection bias of participating schools and one notable difference in characteristics between participating and non-participating schools. Further limitations include that we did not measure the duration of, and adherence to Daily Mile implementation at time of assessment^40^; our previous work shows wide variations in Daily Mile implementation.^21^ Finally, schools in the non-Daily Mile group may be implementing other PA practices which we did not capture.

Although our findings showed no evidence of better health and educational performance attributable to The Daily Mile at the start of primary school, known variations in Daily Mile implementation may have diluted potential impacts. Our longer-term evaluation of The Daily Mile aims to fully understand its benefits. The Daily Mile has an increased uptake in areas of England with high levels of inactivity^13^ and has a global reach. As a whole-school approach, it may contribute towards potential solutions towards limiting disparities and embedding healthy behaviours for lifelong impact.

## Conclusion

Our study did not find any association between doing The Daily Mile and pupils’ PA, mental health and educational performance. However, our longer-term follow-up will allow us to establish whether the inclusion of school-based PA interventions such as The Daily Mile, as part of whole school approach, can contribute to culture and practice to increase pupils’ PA across the primary school years.

## Supplementary material

10.1136/bmjsem-2025-002821 online supplemental file 1

10.1136/bmjsem-2025-002821 online supplemental file 2

10.1136/bmjsem-2025-002821 online supplemental file 3

10.1136/bmjsem-2025-002821 online supplemental file 4

## Data Availability

Data are available upon reasonable request.
