# Supplementary material for: The Daily Mile and children’s physical activity, mental health and educational performance: a quasi-experimental study in Greater London primary schools
Source: BMJ Open Sport Exerc Med. 2026 Jan 3;12(1):e002821. doi: 10.1136/bmjsem-2025-002821 (PMC12766788; doi:10.1136/bmjsem-2025-002821)
Supplement: online supplemental file 2 [file bmjsem-12-1-s002.docx]

**Supplemental material 2**. Characteristics of participating and non-participating schools

|  |  |  | **Participating schools** | | **Non-participating schools** | **All schools** | **p value  (participating vs non-participating schools)** |
| --- | --- | --- | --- | --- | --- | --- | --- |
|  |  |  | **N (%¹)** | | **N (%¹)** | **N (%¹)** |  |
| **Schools, n** | |  | 40 | | 1677 | 1717 |  |
| **Type of school², n (%¹)** | | |  | |  |  |  |
| Academy / Free | | | 5 (13) | | 417 (25) | 422 (25) | 0.07ᵃ |
| Local Authority | | | 35 (88) | | 1260 (75) | 1295 (75) |  |
| **School size (total pupil numbers), n (%¹)** | | | | |  |  |  |
|  | <=500 | | 30 (75) | | 1334 (80) | 1364 (79) | 0.48ᵃ |
|  | >500 | | 10 (25) | | 343 (20) | 353 (21) |  |
| **School pupil gender, n (%¹)** | | |  | |  |  |  |
|  | Mixed | | 40 | | 1671 (100) | 1711 (100) | 0.71ᵃ |
|  | Non-mixed | | - | | 6 (<0) | 6 (<0) |  |
| **Pupils with SEND³, n (%¹)** | | |  | |  |  |  |
|  | <=100 | | 38 (95) | | 1592 (95) | 1630 (95) | 1.00ᵇ |
|  | >100 | | 2 (5) | | 85 (5) | 87 (5) |  |
| **Pupils with English as second language, n (%¹)** | | | |  |  |  |  |
|  | <=100 | | 5 (13) | | 508 (30) | 513 (30) | 0.02ᵃ |
|  | >100 | | 35 (86) | | 1169 (70) | 1204 (70) |  |
| **Pupils eligible for free school meals, n (%¹)** | | | | |  |  |  |
|  | <=100 | | 26 (65) | | 1089 (65) | 1115 (65) | 0.99ᵃ |
|  | >100 | | 14 (35) | | 588 (35) | 602 (35) |  |
| **OFSTED rating⁴, n (%¹)** | | |  | |  |  |  |
| Outstanding/Good | | | 35 (88) | | 1529 (91) | 1564 (91) | 0.42ᵃ |
| Other | | | 5 (13) | | 148 (9) | 153 (9) |  |
| **Area deprivation IDACI⁵ (quintiles), n (%¹)** | | | | |  |  |  |
| (Most deprived) 1 | | | 5 (13) | | 274 (16) | 279 (16) | 0.37ᵃ |
|  | 2 | | 11 (28) | | 432 (26) | 443 (26) |  |
|  | 3 | | 12 (30) | | 315 (19) | 327 (19) |  |
|  | 4 | | 6 (15) | | 399 (24) | 405 (24) |  |
| (Least deprived) 5 | | | 6 (15) | | 257 (15) | 263 (15) |  |
| *All schools at time of study duration; sources: https://www.gov.uk/government/statistics/schools-pupils-and-their-characteristics-january-2021 and https://www.gov.uk/government/collections/statistics-performance-tables | | | | | | | |
| ᵃChi-squared test | | | | | | | |
| ᵇFisher’s exact test | | | | | | | |
| ¹May not total 100% due to rounding | | | | | | | |
| ²Academy/Free Schools are Academy Converter, Academy Sponsor Led and Free Schools; Local Authority Schools are Community, Voluntary Aided, and Voluntary Controlled | | | | | | | |
| ³SEN: Special Educational Needs and Disabilities | | | | | | | |
| ⁴Ofsted: The Office for Standards in Education, Children's Services and Skills; ‘Other’ includes ‘requires improvement’, ‘inadequate’ (rated 'Serious Weaknesses' or 'Special Measures') or rating not available. | | | | | | | |
| ⁵IDACI: Income Deprivation Affecting Children Index | | | | | | | |
