# Supplementary material for: The Daily Mile and children’s physical activity, mental health and educational performance: a quasi-experimental study in Greater London primary schools
Source: BMJ Open Sport Exerc Med. 2026 Jan 3;12(1):e002821. doi: 10.1136/bmjsem-2025-002821 (PMC12766788; doi:10.1136/bmjsem-2025-002821)
Supplement: online supplemental file 3 [file bmjsem-12-1-s003.docx]

**Supplemental material 3**. Sensitivity analysis excluding 4 schools where classes differed in doing The Daily Mile

|  |  | **Daily Mile group** | **Non-Daily Mile group** | **Model 1ᵃ (unadjusted)** | **Model 2 (adjusted)** |
| --- | --- | --- | --- | --- | --- |
|  |  | **mean (sd)*** | **mean (sd)*** | **Difference¹ (95% CI)** | **Difference¹ (95% CI)** |
| **Outcomes** |  |  |  | *(reference = non-Daily Mile group)* | |
| Total MVPA² mins | | ***n=325*** | **n=471** |  |  |
|  |  | 12.0 (12.0) | 9.3 (10.0) | 1.81 (-3.40, 7.61) | -3.08 (-8.71, 2.55)ᵇ |
| Mental health (SDQ)³ | | ***n=442*** | ***n=427*** |  |  |
|  |  | 8.5 (0.4) | 8.8 (0.5) | -0.29 (-1.20, 0.63) | -0.02 (-0.25, 0.21)ᶜ |
| Educational  performance⁴ | Reading | ***n=442*** | ***n=427*** |  |  |
|  |  | 3.2 (0.2) | 3.3 (0.2) | -0.04 (-0.26, 0.18) | -0.02 (-0.25, 0.21)ᶜ |
|  | Writing | ***n=442*** | ***n=427*** |  |  |
|  |  | 3.0 (0.1) | 3.1 (0.2) | -0.04 (-0.24, 0.15) | -0.01 (-0.22, 0.21)ᶜ |
|  | Maths | ***n=442*** | ***n=427*** |  |  |
|  |  | 3.3 (0.2) | 3.3 (0.2) | -0.08 (-0.30, 0.13) | -0.04 (-0.27, 0.19)ᶜ |
| *Postestimation command *predict* used to determine mean and sd | | | | | |
| ᵃModel 1 includes random effects to allow for clustering at school and class level | | | | | |
| ᵇFully adjusted Model 2 includes sex, ethnic group, IDACI and month of assessment | | | | | |
| ᶜFully adjusted Model 2 includes adjustments for sex, ethnic group and IDACI | | | | | |
| ¹Difference in total MVPA between the Daily Mile pupils compared with non-Daily Mile pupils | | | | | |
| ²MVPA: moderate-to-vigorous physical activity | | | | | |
| ³Mental health as measured by the Strengths and Difficulties Questionnaire (SDQ) reported by parents. Total difficulties score is sum of scores on 4/5 SDQ subscales; scores range from 0-40. | | | | | |
| ⁴Age related expectations; reported by class teacher; scale from 0 (below expected) to 4 (above expected) | | | | | |
