# Supplementary material for: The Daily Mile and children’s physical activity, mental health and educational performance: a quasi-experimental study in Greater London primary schools
Source: BMJ Open Sport Exerc Med. 2026 Jan 3;12(1):e002821. doi: 10.1136/bmjsem-2025-002821 (PMC12766788; doi:10.1136/bmjsem-2025-002821)
Supplement: online supplemental file 4 [file bmjsem-12-1-s004.docx]

**Supplemental material 4.** Sensitivity analysis including BMI as a covariate to the fully adjusted model

|  |  |  | **Model 1ᵅ (unadjusted) Difference (95% CI)** | | **Model 2ᵇ (adjusted) Difference (95% CI)** |
| --- | --- | --- | --- | --- | --- |
|  |  |  | *(reference = non-Daily Mile group)* | | |
| **MVPA minutes**¹ | | | 1.68 (-1.17, 4.53) | | 0.84 (-2.13, 3.81) |
| **Sex** *(reference = females)* | | |  | | 1.17 (-0.06, 2.39) |
| **Ethnic group** *(reference = white)* | | |  | |  |
|  | Mixed | |  | | 1.10 (-0.79, 2.98) |
|  | Asian | |  | | 1.65 (-0.10, 3.40) |
|  | Black | |  | | 0.12 (-2.15, 2.39) |
|  | Other | |  | | 1.81 (-1.81, 5.42) |
| **IDACI quintiles** *(reference = 1 (most deprived) borough)* | | | |  |  |
| 2 | | |  | | 7.45 (-2.35, 17.26) |
|  |  | 3 |  | | 6.94 (-3.16, 17.04) |
|  |  | 4 |  | | 2.55 (-8.11, 13.21) |
| 5 (least deprived) | | |  | | 11.01 (0.18, 21.83)³ |
| **Month of assessment**² *(reference = Jan)* | | | | |  |
| Feb | | |  | | -1.69 (-11.58, 8.19) |
|  | Mar | |  | | 1.32 (-8.27, 10.91) |
|  | Apr | |  | | -2.25 (-14.76, 10.27) |
|  | May | |  | | 0.20 (-9.12, 9.53) |
|  | Jun | |  | | 1.42 (-7.78, 10.62) |
|  | Jul | |  | | 4.37 (-8.04, 16.77) |
|  | Oct | |  | | 17.12 (-2.82, 37.06) |
|  | Nov | |  | | -1.88 (-11.58, 7.81) |
|  | Dec | |  | | 5.22 (-8.19, 18.63) |
| **BMI percentile** |  | |  | | 0.02 (0.00, 0.04)³ |
| ᵅModel 1 includes random effects for clustering at day, pupil, class, and school level | | | | | |
| ᵇModel 2 includes adjustments for sex, ethnic group, IDACI, month of assessment, and BMI percentile (continuous) | | | | | |
| ¹Total MVPA minutes and number of days per child with full school hours data | | | | | |
| ²No assessments took place during the months of August or September | | | | | |
| ³Significant at <0.05 p level | | | | | |
